# Radiation Doses to the Entire Catheterization Laboratory Team With a Novel Radiation Protection Device

**DOI:** 10.1016/j.jscai.2023.101109

**Published:** 2023-10-19

**Authors:** Malav J. Parikh, Lahdan Refahiyat, Timothy A. Joseph, David McNamara, Ryan D. Madder

**Affiliations:** Frederik Meijer Heart & Vascular Institute, Corewell Health, Grand Rapids, Michigan

**Keywords:** occupational health, Protego, radiation safety

## Abstract

**Background:**

A novel radiation protection system has recently been shown to shield the primary operator from scatter radiation, but whether it shields other members of the catheterization laboratory team remains unknown.

**Methods:**

Radiation exposure data were collected prospectively in 50 coronary angiography cases, in which 25 were completed using standard radiation protection and 25 with a novel system consisting of a series of rigid shields and flexible radiation-resistant drapes. Radiation doses, measured with real-time dosimeters, were compared between the 2 groups.

**Results:**

There were no significant differences between groups with respect to patient or procedural characteristics, including air kerma (*P* = .97) and dose area product (*P* = .17). The primary operator received a median head-level radiation dose of 0.0 [0.0, 0.0] μSv with the novel radiation protection system and 2.1 [0.7, 3.3] μSv with standard radiation protection (*P* < .001). Scrub technologists had a median head-level radiation dose of 0.0 [0.0, 0.0] μSv with the novel radiation protection system and 0.3 [0.1, 0.4] μSv with standard radiation protection (*P* < .001). The median head-level radiation dose among circulating nurses was 0.0 [0.0, 0.0] μSv with the novel radiation protection system and was 0.1 [0.0, 0.2] μSv with standard radiation protection (*P* < .001).

**Conclusions:**

Compared to standard radiation protection with lead aprons, use of a novel radiation protection system during coronary angiography was associated with significantly lower head-level radiation doses among all members of the catheterization laboratory team.

## Highlights

• A novel radiation protection system was developed to enhance radiation safety.
• The system was associated with lower radiation doses than standard leaded aprons.
• With the system, median head-level doses were 0.0 μSv among physicians and staff.

## Central Illustration

**Figure:**
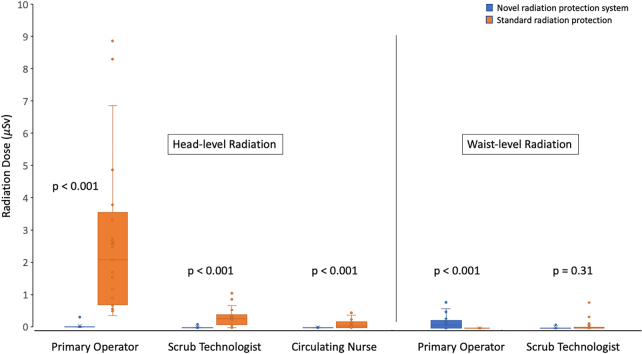

## Introduction

Radiation exposure in the cardiac catheterization laboratory is an occupational hazard to operators and staff members and has been linked to a variety of acquired conditions including premature cataracts, vascular injury, certain cancers, and skin injuries.^1^, ^2^, ^3^, ^4^ The traditional protection method of wearing leaded aprons does not provide full body protection and typically leaves the head, arms, and lower legs uncovered. In addition, prolonged use of leaded aprons likely carries a risk of orthopedic injury, which represents a major occupational hazard among interventional cardiologists and other team members.^5^ These prior studies highlight the need for novel strategies to minimize radiation exposure while simultaneously reducing the risk of orthopedic injury to catheterization laboratory personnel.

A novel radiation protection system has recently been developed to substantially lessen occupational radiation exposure and potentially eliminate the need to wear leaded aprons for all procedural personnel in the catheterization laboratory.^6^ This system consists of a combination of interconnecting leaded shields placed around and below the patient and procedure table in order to protect laboratory personnel from scatter radiation. A recent preclinical study showed this system to be associated with significantly lower scatter radiation doses at multiple locations in the procedure room.^6^ Two recent clinical studies using this novel system showed that its use was associated with strikingly low radiation doses to the primary operator, concluding that this system may eliminate the need of the primary operator to wear lead.^7^^,^^8^ However, the performance of this novel radiation protection system has not yet been evaluated in the clinical setting for its ability to protect all members of the procedural team from scatter radiation. The present study was performed to evaluate radiation doses to the primary operator, scrub technologist, and circulating nurse during invasive coronary angiography procedures performed with the novel radiation protection system.

## Methods

### Study design

This single-center prospective observational study was designed to investigate radiation doses to the primary operator, scrub technologist, and circulating nurse during coronary angiography procedures performed using a novel radiation protection system. Radiation exposure data were collected prospectively in 50 coronary angiography cases, in which 25 consecutive cases were performed using the novel system, and 25 consecutive cases using standard radiation protection wearing leaded aprons. All cases were performed in a single fluoroscopy suite using a state-of-the art fluoroscopy system (AlluraClarity, Philips) with real-time image noise reduction technology (Clarity IQ, Philips). In all cases, fluoroscopy was performed at a frame rate of 7.5 frames per second. Fluoroscopy and cineangiography were utilized according to operator discretion. The study was conducted as a quality improvement initiative to determine whether use of the system was associated with low enough radiation doses to warrant further consideration of future clinical use of the system at the study institution, and as a result, approval by the local institutional review board was not required.

### Standard radiation protection

In the control group, standard radiation protection measures were employed in all cases. According to institutional standards, 2 shields were positioned between the patient and operating physician: a ceiling-mounted upper body lead shield with a patient contour cutout, and a lower body lead shield attached to the side of the operating table extending from table to floor. A radiation-absorbing disposable pad (RadPad, Worldwide Innovations & Technologies) was utilized in all cases.

### Novel radiation protection system

**Figure 1:**
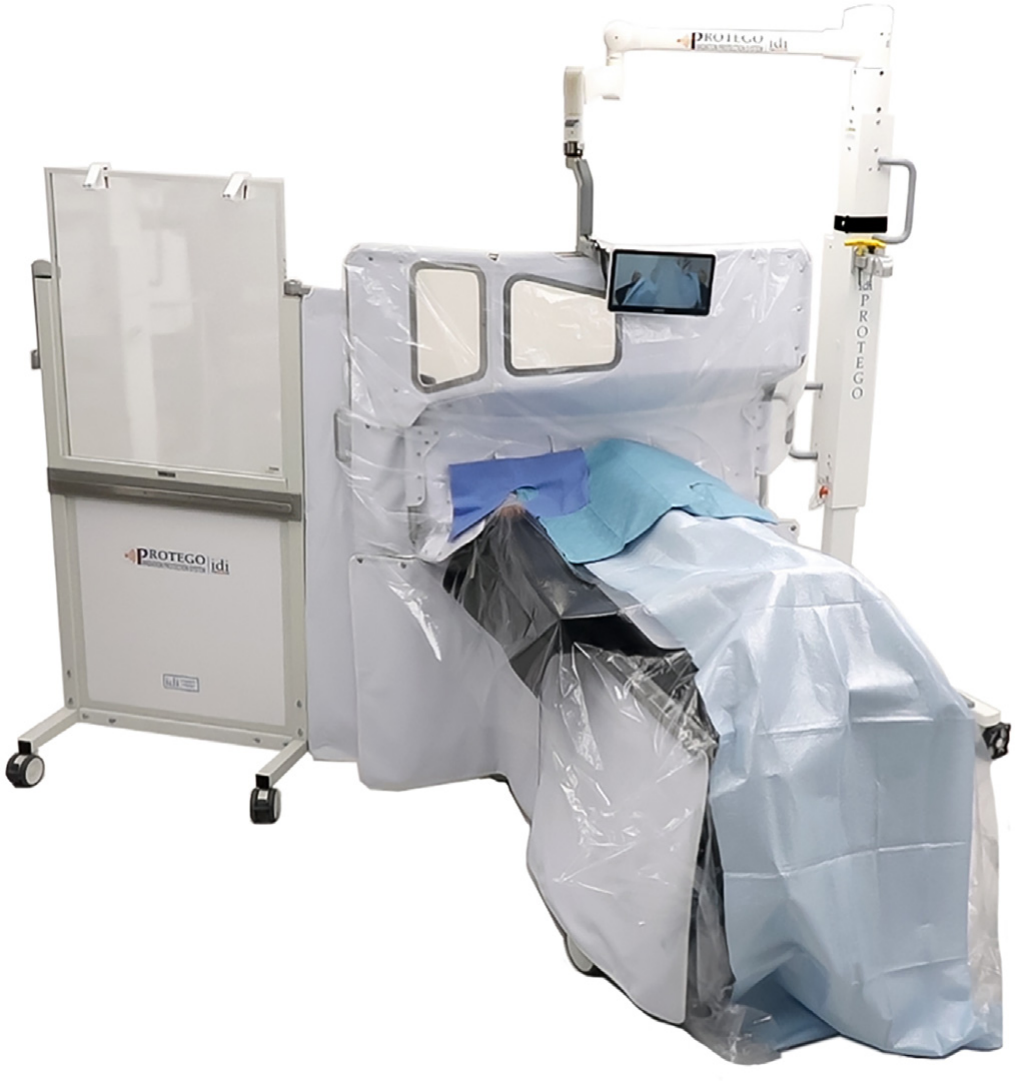
**Novel radiation protection system.** Shown is the radiation protection system (Protego, Image Diagnostics) evaluated in this study. The system consists of a combination of rigid shields above and below the table, as well as interconnecting flexible radiation-resistant drapes.

The radiation protection system (Protego, Image Diagnostics) evaluated in this study consists of a combination of rigid shields above and below the table as well as interconnecting flexible radiation-resistant drapes (Figure 1). As described previously by Dixon et al,^6^ the key elements of the protection system include: (1) an upper shield located above the table with an angulated design to passively accommodate unimpeded C-arm motion; (2) a lower shield located below the table; (3) an accessory side shield that affixes to the operators’ side of the system and extends the umbrella of protection; (4) flexible radiation-resistant drapes that interconnect to the fixed shields and overlap with similar drapes with portals for vascular access; and (5) disposable sterile drapes that cover the fixed and flexible components.

### Radiation dose metrics

The primary measure of interest in this study was the radiation dose received by the primary operator, scrub technologist, and circulating nurse. Radiation doses were prospectively collected using a commercially available real-time dosimetry system (RaySafe i2, Unfors RaySafe) and are reported as the personal dose equivalent (H_p_(10)) measured in μSv. The primary operator and scrub technologist each wore dosimeters at the head and waist level. The circulating nurse wore a dosimeter at the head level. Head-level dosimeters were worn on the left anterior side of the thyroid collar or on the left anterior side of glasses, external to any leaded covering. Waist-level dosimeters in the arm with the novel system were worn on the left anterior side of the body at waist level outside of any leaded coverings, in order to generate data on doses that would be received at the waist level if no lead aprons were worn while using the novel radiation protection system. The waist-level dosimeter in the control arm was worn inside of the leaded apron. Radiation doses measured by each dosimeter were recorded at the completion of each case. Estimates of patient radiation doses, which included fluoroscopy time, air kerma, and dose area product, were automatically calculated by the fluoroscopy imaging system and recorded after each case.

### Statistical analysis

Descriptive statistics were used to summarize all variables. Shapiro-Wilk test for normality was performed to determine the presence of a normal distribution. Normally distributed continuous variables are shown as mean ± standard deviation. Nonnormally distributed continuous variables are shown as median [25th percentile, 75th percentile]. Categorical variables are shown as count (% frequency). Statistical analyses of nonnormally distributed variables were performed using Mann-Whitney *U* test. Statistical analyses of normally distributed variables were performed with *t* test. An alpha level of .05 was used.

## Results

Radiation exposure data were prospectively collected in 25 consecutive coronary angiography cases performed with the novel radiation protection system and in 25 consecutive cases with standard protection. A summary of patient characteristics and procedural details is presented in Table 1. In brief, radial access was used in 88.0% of cases in both groups (*P* = 1.0), and there were no significant differences between groups with respect to patient age, body mass index, fractional flow reserve, or percutaneous coronary intervention (PCI). Performance of bypass graft angiography was performed in 12.0% of cases with the novel protection system and in 4.0% of cases with standard protection (*P* = .30). No procedural complications occurred in any of the cases in either group.

**Table 1:** Patient and procedural characteristics among coronary angiography cases performed with a novel radiation protection system vs standard radiation protection

	Novel radiation protection system (n = 25)	Standard radiation protection (n = 25)	*P*
Age, y	72 [62, 77]	63 [59, 69]	.07
Male	17 (68.0)	18 (72.0)	.76
Body mass index, kg/m^2^	33.6 ± 6.5	30.4 ± 6.2	.08
Arterial access site			>.99
Right radial	22 (88.0)	22 (88.0)	
Right femoral	3 (12.0)	3 (12.0)	
Left heart catheterization	17 (68.0)	17 (68.0)	>.99
Fractional flow reserve	3 (12.0)	3 (12.0)	>.99
Bypass graft angiography	3 (12.0)	1 (4.0)	.30
Percutaneous coronary intervention	1 (4.0)	0 (0.0)	.31

Age is shown as median [25th percentile, 75th percentile]. Body mass index is shown as mean ± SD. All other variables are shown as n (%).

### Patient radiation dose metrics

Patient radiation doses metrics for both groups are summarized in Table 2. No significant differences were identified between groups for fluoroscopy time (*P* = .12), air kerma (*P* = .97), or dose area product (*P* = .17).

**Table 2:** Case time and patient radiation dose metrics.

	Novel radiation protection system (n = 25)	Standard radiation protection (n = 25)	*P*
Case time, min	19.5 [15.8, 27.3]	18.0 [12.0, 23.0]	.052
Fluoroscopy time, min	5.4 [3.5, 7.2]	4.0 [3.0, 4.9]	.12
Air kerma, mGy	244 [178, 382]	235 [206, 298]	.97
Dose area product, mGy × cm^2^	15.9 [11.5, 19.3]	12.2 [10, 15.6]	.17

Values shown are median [25th percentile, 75th percentile].

### Primary operator radiation dose

Physician and staff member radiation doses are summarized in the Central Illustration and in Table 3. The primary operator received a median head-level radiation dose of 0.0 [0.0, 0.0] μSv with the novel radiation protection system and 2.1 [0.7, 3.3] μSv with standard radiation protection (*P* < .001). The primary operator head-level radiation dose was 0.0 μSv in 24 (96.0%) cases with the novel system. In the single case in which the primary operator received measurable head-level radiation, the dose was 0.3 μSv. In contrast, primary operator head-level radiation was detected in all (100%) cases in the standard radiation protection group. The cumulative head-level radiation dose was 0.3 μSv across 25 cases performed with the novel radiation protection system and 65.1 μSv across 25 cases performed with standard radiation protection.

**Central Illustration:**
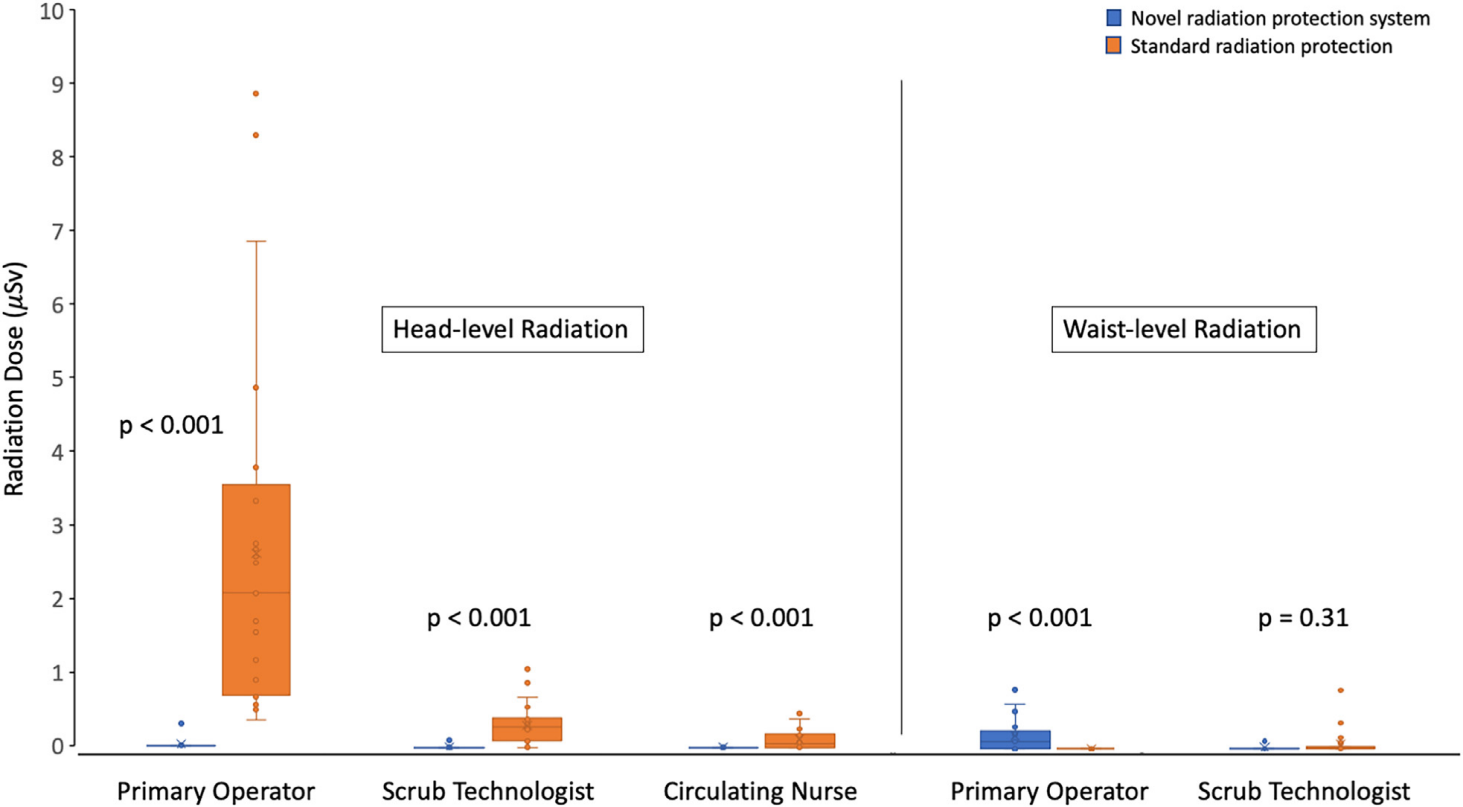
Radiation doses using a novel radiation protection system vs standard radiation protection. Shown are box and whisker plots of head-level and waist-level radiation doses received during coronary angiography cases performed using a novel radiation protection system (blue) and with standard radiation protection (orange).

**Table 3:** Physician and staff radiation doses.

Dosimeter location	Novel system (μSv)	Control (μSv)	*P*
Primary operator			
Head-level median dose	0.0 [0.0, 0.0]	2.1 [0.7, 3.3]	<.001
Head-level min dose	0.0	0.3	
Head-level max dose	0.3	8.9	
Waist-level median dose	0.1 [0.0, 0.2]	0.0 [0.0, 0.0]	<.001
Waist-level min dose	0.0	0.0	
Waist-level max dose	0.8	0.0	
Scrub technologist			
Head-level median dose	0.0 [0.0, 0.0]	0.3 [0.1, 0.4]	<.001
Head-level min dose	0.0	0.0	
Head-level max dose	0.1	1.1	
Waist-level	0.0 [0.0, 0.0]	0.0 [0.0, 0.0]	.31
Waist-level min dose	0.0	0.0	
Waist-level max dose	0.1	0.8	
Circulating nurse			
Head-level median dose	0.0 [0.0, 0.0]	0.1 [0.0, 0.2]	<.001
Head-level min dose	0.0	0.0	
Head-level max dose	0.0	0.5	

Values in brackets are [25th percentile, 75th percentile].

max, maximum; min, minimum.

The primary operator received a median waist-level radiation dose of 0.1 [0.0, 0.2] μSv with the novel radiation protection system, and 0.0 [0.0, 0.0] μSv with standard radiation protection (*P* < .001). The recorded waist-level dose was 0.0 μSv in 9 (36.0%) cases with use of the novel radiation protection system and in all (100%) cases with standard radiation protection. With use of the novel radiation protection system, the highest recorded waist-level dose for the primary operator was 0.8 μSv.

### Scrub technologist radiation dose

Radiation doses for the scrub technologist were only available in 24 of the 25 cases with the novel system, as there was a single case in this group where the scrub technologist did not wear the dosimeters. The scrub technologist had a median head-level radiation dose of 0.0 [0.0, 0.0] μSv with the novel radiation protection system and 0.3 [0.1, 0.4] μSv with standard radiation protection (*P* < .001). The scrub technologist head-level radiation dose was 0.0 μSv in 23 of 24 (95.8%) cases with the novel system and in 3 of 25 (12.0%) cases with standard radiation protection. In the single case in which the scrub technologist received measurable head-level radiation with the novel radiation protection system, the dose was 0.1 μSv. The cumulative head-level radiation dose was 0.1 μSv across 24 cases performed with the novel radiation protection system and was 7.4 μSv across 25 cases performed with standard radiation protection.

The median waist-level radiation dose for the scrub technologist was 0.0 [0.0, 0.0] μSv in both groups (*P* = .31). With use of the novel radiation protection system, no detectable radiation was observed at the waist level in 21 cases (87.5%). The 3 cases with the novel system with detectable waist-level radiation each were characterized by a dose of 0.1 μSv.

### Circulating nurse radiation dose

The median head-level radiation dose among circulating nurses was 0.0 [0.0, 0.0] μSv with the novel radiation protection system and was 0.1 [0.0, 0.2] μSv with standard radiation protection (*P* < .001). The recorded head-level radiation dose among circulating nurses was 0.0 μSv in all 25 (100%) cases with the novel radiation protection system and in 8 of 25 (32.0%) cases with standard radiation protection.

## Discussion

When compared with standard radiation protection, use of a novel radiation protection system during coronary angiography in the present study was associated with low radiation doses among all members of the catheterization laboratory team. Not only were head-level radiation doses significantly lower relative to those observed with standard radiation protection among the physician, scrub technologists, and circulating nurses, but the absolute head-level doses with use of the novel radiation protection system were exceptionally low. Accordingly, the observed median head-level radiation doses with use of the novel protection system were 0.0 μSv for the primary operator, scrub technologist, and nurse. Furthermore, no head-level radiation was detected in 96% of cases for the primary operator and scrub technologist and in 100% of cases among circulating nurses. Waist-level radiation doses were also observed to be low, with median values of 0.1 μSv and 0.0 μSv among operating physicians and scrub technologists, respectively. It is important to note that the median waist-level dose of 0.1 μSv for the primary operator was significantly higher than that observed with standard radiation protection because no waist-level radiation was detected in any case underneath the lead apron of the primary operator in the standard radiation protection arm. However, the significance of a median waist-level dose of 0.1 μSv per case remains uncertain, especially when interpreted in the context of an annual occupational dose limit of 50,000 μSv. We postulate that the source of the observed waist-level exposure is likely related to gaps in lead coverage at the arterial access site and that further development of the novel radiation protection system may eventually mitigate this “leak” in future iterations of the device. Finally, use of the novel radiation protection system was not at the expense of higher patient radiation doses, as no significant differences were observed between groups with respect to fluoroscopy time, air kerma, or dose area product.

The results of the present study are consistent with those recently reported by Rizik et al,^8^ in which use of the novel radiation protection system was associated with significantly lower radiation doses to the primary operator. The present results build upon those of Rizik et al by demonstrating novel radiation protection system is associated with lower radiation doses among all members of the catheterization laboratory team. This may represent an advantage over other radiation protection devices, including robotic systems and suspended lead suits, wherein the radiation protection is mostly focused on the primary operator. Indeed, protection of the entire catheterization laboratory team is of paramount importance considering the occupational risks of working in the catheterization laboratory are not limited to physicians but also impact scrub technologists and nurses.^1^, ^2^, ^3^

### Occupational radiation doses in the catheterization laboratory

The head-level radiation doses observed with use of the novel radiation protection system in this study were significantly lower those in the standard radiation protection arm and were also considerably lower than doses previously reported in the contemporary era. In a prior study evaluating head-level radiation doses among physicians wearing traditional leaded aprons, median doses of 7.1 μSv during diagnostic coronary angiography and 14.3 μSv during PCI were reported.^9^ Furthermore, much higher head-level doses are likely commonplace in contemporary practice, as 25% of cases in this prior study had physician head-level radiation doses of >26.7 μSv and >57.2 μSv in diagnostic coronary angiography and PCI cases, respectively.^9^ Similarly, a median dose of 56 μSv was recently shown at the thyroid level with standard radiation protection among physicians performing cases in a modern catheterization laboratory.^8^ In contrast, use of the novel radiation protection system in the present study was associated with a median head-level dose of 0.0 μSv among physicians, and no head-level radiation was detected in 96% of cases. In the single case in which the physician did receive measurable head-level radiation, the dose was only 0.3 μSv.

Similar to the low radiation doses observed among physicians in this study, use of the novel radiation protection system was associated with median radiation doses of 0.0 μSv among scrub technologists and circulation nurses. The observation that no head-level radiation was detected among circulating nurses in 25 consecutive cases in this study is encouraging, as a previous study reported median head-level radiation doses among circulating nurses of 0.2 μSv in diagnostic coronary angiography and 2.1 μSv in PCI.^10^

### Occupational risks

Chronic radiation exposure in the catheterization laboratory may pose a risk for the development of certain malignancies, cataracts, and premature carotid atherosclerosis.^1^, ^2^, ^3^, ^4^ The risks posed by occupational radiation has been highlighted by reports that scatter radiation from single endovascular procedures is associated with a measurable rise and fall of DNA damage biomarkers.^11^ The risks of working in the catheterization laboratory also include the risk of chronic orthopedic injury attributable to wearing heavy lead apparel to mitigate radiation exposure. This practice has led to both orthopedic injuries and work-related chronic pain.^5^^,^^12^^,^^13^ The ideal solution to addressing these dual occupational risks would simultaneously reduce radiation exposure and eliminate the need to wear leaded aprons.

Other radiation protection systems have been developed to address the occupational hazards of working in the catheterization laboratory but have primarily been directed at the operating physician. Accordingly, suspended lead suits offer superior radiation protection to the primary operator compared to traditional lead aprons, with one study showing median head-level radiation doses of 0.2 μSv in suspended lead suits as compared to 10.2 μSv in traditional lead aprons.^9^ Unlike the novel radiation protection system evaluated in the present study, suspended lead suits do not afford protection to a physician’s hands and forearms. A robotic system for performing PCI can protect the primary operator from radiation without the need for wearing leaded aprons.^14^ It is important to recognize that both the suspended lead suit and the robotic PCI system only shield the primary operator from radiation, while remaining procedural staff are relegated to continuing with traditional lead aprons. In contrast, the radiation protection system evaluated in the present study seemingly affords radiation protection to all members of the catheterization laboratory team and has the potential to eliminate the need to wear leaded aprons.

### Limitations

The present pilot study is limited by its small size and single-center observational design. Studies conducted at multiple centers in a larger number of patients are needed. It should be noted that the low radiation doses observed in this study were achieved with meticulous attention to detail to avoid gaps in shielding when setting up the system. Whether these results can be replicated across a greater series of cases requires further evaluation. The doses achieved in this study were achieved with a state-of-the-art fluoroscopy system. Performance of the shielding system with use of older fluoroscopy equipment has yet to be determined. A chief limitation of this study is the small number of PCI cases performed, as only a single PCI was performed in each group. Considering radiation doses are typically higher with PCI compared to diagnostic cases, extrapolation of these data to PCI cases should be done with caution, and additional studies in the setting of PCI are needed. Importantly, 2 recent studies have demonstrated low radiation doses with the novel radiation protection system in a larger series of PCI cases.^7^^,^^8^ The fact that no left radial access cases were performed represents an additional limitation. Finally, we recognize that information regarding the cost of the novel radiation protection system, which is not reported in this manuscript, is relevant to catheterization laboratories when considering the possible acquisition of a system to enhance their radiation protection.

## Conclusions

The use of a novel radiation shielding system during coronary angiography was associated with low radiation doses among all members of the catheterization laboratory team. Additional studies of this system are required to determine if the system will ultimately allow staff to perform cases safely without wearing protective lead apparel.
